# Intracerebral Distribution of the Oncometabolite d-2-Hydroxyglutarate in Mice Bearing Mutant Isocitrate Dehydrogenase Brain Tumors: Implications for Tumorigenesis

**DOI:** 10.3389/fonc.2016.00211

**Published:** 2016-10-11

**Authors:** Amanda J. Pickard, Albert S. W. Sohn, Thomas F. Bartenstein, Shan He, Yi Zhang, James M. Gallo

**Affiliations:** ^1^Department of Pharmacology and Systems Therapeutics, Icahn School of Medicine at Mount Sinai, New York, NY, USA; ^2^Fels Institute for Cancer Research and Molecular Biology, Philadelphia, PA, USA; ^3^Department of Microbiology and Immunology, Temple University, Philadelphia, PA, USA

**Keywords:** oncometabolite brain distribution, immune function, transport model, glymphatic clearance, brain microdialysis

## Abstract

The prevalence of mutant isocitrate dehydrogenase 1 (IDH1) brain tumors has generated significant efforts to understand the role of the mutated enzyme product d-2-hydroxyglutarate (D2HG), an oncometabolite, in tumorigenesis, as well as means to eliminate it. Glymphatic clearance was proposed as a pathway that could be manipulated to accelerate D2HG clearance and dictated the study design that consisted of two cohorts of mice bearing U87/mutant IDH1 intracerebral tumors that underwent two microdialysis – providing D2HG interstitial fluid concentrations – sampling periods of awake and asleep (activate glymphatic clearance) in a crossover manner. Glymphatic clearance was found not to have a significant effect on D2HG brain tumor interstitial fluid concentrations that were 126.9 ± 74.8 μM awake and 117.6 ± 98.6 μM asleep. These concentrations, although low relative to total brain tumor concentrations of 6.8 ± 3.6 mM, were considered sufficient to be transported by interstitial fluid and taken up into normal cells to cause deleterious effects. A model of D2HG CNS distribution supported this contention and was further supported by *in vitro* studies that showed D2HG could interfere with immune cell function. The study provides insight into the compartmental distribution of D2HG in the brain, wherein the interstitial fluid serves as a dynamic pathway for D2HG to enter normal cells and contribute to tumorigenesis.

## Introduction

The identification of *isocitrate dehydrogenase 1* (*IDH1*) gene mutations in more than 70% of low grade astrocytomas and oligodendrogliomas as well as secondary glioblastomas (GBMs) (1, 2) has led to a significant drive to develop diagnostic and treatment modalities able to exploit this mutation for therapeutic gain. The most common mutation, the heterozygous substitution of a guanine for an adenine nucleobase at codon 395 (G395A), results in an R132H mutation in the active site of IDH1 (3). This mutation results in a heterodimer containing wild-type and mutant IDH1 (mIDH1) subunits, allowing for both the oxidative decarboxylation of isocitrate to α-ketoglutarate (KG) and the reduction of KG to d-2-hydroxyglutarate (D2HG), an oncometabolite (4).

D2HG has been shown to accumulate in patient-derived tumors in millimolar concentrations (4); however, its precise biological role is the subject of ongoing investigations. It is implicated in methylome reprogramming – both DNA and histones – of cancers by inhibiting histone demethylases and the TET family of hydrolases resulting in the CpG island methylator phenotype (G-CIMP) (5–7). The enantiomer of D2HG, l-2-hydroxyglutarate (L2HG), has been shown to be a more potent inhibitor of the KG-dependent dioxygenases (8, 9), and its increased production has been correlated with hypoxic tumor microenvironments (10, 11). Both enantiomers are present in bodily fluids as a by-product of metabolism, but their concentrations are regulated by stereospecific dehydrogenase enzymes capable of oxidation back to KG (12, 13). The high concentrations of D2HG in brain tumors and the aforementioned biological actions of this neomorphic metabolite are implicated in tumorigenesis, although the potential effects on normal cells, including those involved in immunological responses remain to be defined.

The high concentrations of D2HG produced in mIDH1 brain tumors overcome detoxification by d-2-hydroxyglutarate dehydrogenase (D2HGDH), and thus, other avenues to minimize D2HG are avidly being pursued. First and foremost are the use of mIDH1 enzyme inhibitors, such as AG120 or AG881, that are currently being evaluated in the clinic for advanced hematologic cancers, including acute myeloid leukemia, as well as GBMs (2, 14, 15). The glymphatic system, a newly discovered mechanism of CNS clearance for proteins associated with neurodegenerative diseases (16), may be an alternate means to accelerate D2HG elimination from the brain. This system, which is tightly regulated by sleep/wake cycles and aquaporin-4 expression, was shown to increase β-amyloid clearance through pharmacological means – both anesthesia and an adrenergic receptor blocking cocktail – that simulate sleep (17). Since very little is known about the brain disposition of D2HG, including its potential as a biomarker, its role in tumorigenesis, and its propensity for glymphatic clearance, we monitored D2HG and L2HG concentrations in brain tumor interstitial fluid (BTIF) by performing microdialysis in both awake and anesthetized mice bearing intracerebral mIDH1 tumors.

## Materials and Methods

### Chemicals and Reagents

All chemicals and reagents were purchased from commercial sources and used without further purification. L2HG disodium salt and (+)-*O*,*O*′-diacetyl-l-tartaric anhydride (DATAN) were obtained from Sigma-Aldrich (St. Louis, MO, USA). D2HG disodium salt and dl-2-hydroxyglutaric acid-d_3_ (dl-2HG-d_3_) disodium salt were obtained from Cayman Chemical (Ann Arbor, MI, USA) and Toronto Research Chemicals (Toronto, ON, Canada), respectively. Guide cannulas and microdialysis probes (Meta-Quant) were purchased from Brainlink B.V. (Groningen, Netherlands). Mass spectrometry-grade chemicals and solvents were obtained from Fisher Scientific (Fair Lawn, NJ, USA) or Sigma-Aldrich (St. Louis, MO, USA). All other chemicals and materials were obtained from commercial suppliers.

### Animals and Cell Lines

Male NCr nude mice (nu/nu, 4 weeks old) were purchased from Taconic Farms (Germantown, NY, USA). All animal experiments were approved by the Institutional Animal Care and Use Committee according to the National Institutes of Health guidelines. U87 cells that had been stably transfected with the R132H IDH1 mutation were kindly provided by Agios Pharmaceuticals (Dr. Michael Su, Cambridge, MA, USA), and used as previously described (4). R132H_mIDH1/U87 cells were grown as a monolayer in Dulbecco’s modified Eagle’s medium with 10% fetal bovine serum, 1× penicillin and streptomycin, and 300 μg/mL of G418 selection antibiotic and maintained in a humidified atmosphere of 5% CO_2_ in air at 37°C.

### Tumor Implantation

Male NCr nude mice (5 weeks old) were anesthetized using an intraperitoneal dose (0.1 mL/10 g body weight) of 10% ketamine hydrochloride (100 mg/mL) and 8% xylazine (20 mg/mL) in water and placed in a stereotaxic frame. Tumor cells were prepared fresh from culture and kept in suspension (1 × 10^5^ cells/μL in phosphate-buffered saline) on ice until implantation. Each mouse had 3 μL of suspension injected (3 × 10^5^ tumor cells) into the right caudate putamen (0.7 mm anterior and 2.2 mm lateral from the bregma) at a depth of 2.5 mm. The injection site on the skull was sealed with sterile bone wax, and the incision was closed and fixed with wound glue. Mice received a standard mouse diet and water *ad libitum* and received 3 days of post-operative analgesic care that consisted of a daily 5-mg/kg subcutaneous dose of carprofen. Animals were weighed, assessed for body condition score, and monitored for neurological symptoms daily.

### Guide Cannula Implantation

Thirteen days after tumor implantation (corresponding to at least 7 days before the microdialysis studies), guide cannulas were implanted. Mice were again anesthetized and placed in the stereotaxic frame in the same manner as above. Incision sites from the first surgery were opened, and the injection site located on the skull. A guide cannula was implanted at the injection site and secured with two anchor screws and dental cement. Mice were then individually housed for the duration of the study and received a standard mouse diet and water *ad libitum* and received 3 days of post-operative analgesic care that consisted of a daily 5-mg/kg subcutaneous dose of carprofen. Animals were weighed, assessed for body condition score, and monitored for neurological symptoms daily.

### Brain Microdialysis

Microdialysis studies were completed 20–23 days after glioma cell implantation. This generally coincided with either a 20% loss in body weight, the first sign of neurological symptoms, or an observation of a body condition score of 2 or lower. For microdialysis, a harness was secured around the mouse’s torso, and the dummy probe from the guide cannula was removed and replaced with a brain microdialysis probe that contains two inlets; one referred to as the make-up flow rate and the other the low flow rate inlet that perfused the dialysis membrane. The probes were perfused at a total flow rate of 1 μL/min (both inlets initially at 0.5 μL/min from each pump) for at least 20 min with sterile water and artificial cerebrospinal fluid (147 mM NaCl, 2.7 mM KCl, 1.2 mM CaCl_2_, and 0.85 mM MgCl_2_). Flow rates were then adjusted as the probes were continued to be perfused for an additional 40 min at 0.9 μL/min with sterile water from the make-up pump and 0.1 μL/min with artificial cerebrospinal fluid from the low flow rate pump. After this equilibration period, the study commenced with brain microdialysis samples collected in 20-min intervals into a CMA (Harvard Apparatus, Holliston, MA, USA) 470 fraction collector that maintained the samples at 4–6°C for the duration of the study. Samples were transferred to sterile 200 μL polypropylene tubes after collection and stored at −80°C until they were analyzed.

Mice were randomly separated into the awake–asleep and asleep–awake cohorts. Mice that were awake first had brain microdialysis performed for 2 h, then were placed under anesthesia (1 L/min O_2_, 2.5% isoflurane for induction, 1 L/min O_2_, 1% isoflurane for maintenance). Sampling continued for an additional 2 h. Mice that were under anesthesia first were placed under anesthesia (same as in the other cohort), but immediately after the equilibration period of the probe. After a 2-h asleep period, the anesthesia was stopped, and a 1-h recovery period commenced prior to a 2-h awake sampling period. All mice regained consciousness by the end of the recovery period. At the conclusion of microdialysis, brain, tumor, and plasma were collected.

### Plasma Collection

At the conclusion of brain microdialysis, with mice under heavy anesthesia (1 L/min O_2_, 2.5% isoflurane), cardiac puncture was performed to obtain a whole blood sample using a 1-mL syringe and 23-G needle. Total recovered volume was immediately injected into a K_2_EDTA-coated tube. The tube was inverted at least 12 times to ensure that all of the blood was exposed to anticoagulant, and then the sample was stored on ice until processing. The mouse was euthanized by cervical dislocation while under isoflurane anesthesia. Whole blood with anticoagulant was transferred to a 1.5- or 0.5-mL microcentrifuge tube as appropriate and centrifuged at 3000 rpm and 4°C for 15 min. Supernatant (plasma) was carefully removed from tube while leaving pellet and Buffy coat undisturbed and transferred to new 0.5-mL Eppendorf tubes in 100 μL aliquots, labeled, and stored at −80°C until analyzed.

### Brain and Tumor Homogenization

Whole brain and tumor were harvested from each mouse. Indentation of the microdialysis probe was checked to ensure proper sampling location in the tumor. Brain and tumor samples were separated into three sections: tumor, brain (ipsilateral to tumor), and brain (contralateral to tumor). Sections were rinsed with ice cold sterile water and stored on ice until they were weighed. Each section was transferred to a sample tube to be weighed. For every gram of tissue present, 5 mL of ice cold sterile water was added, resulting in a final concentration of 200 mg tissue/mL. Homogenization was performed using a PolyTron PT 2100 tissue homogenizer set to 30,000 rpm for 5 s. The homogenizer was cleaned before and after each tissue sample. Homogenates were transferred to labeled 1.5-mL microcentrifuge tubes and stored at −80°C until ready to be analyzed.

### Derivatization and Sample Preparation

#### Microdialysis Samples

A 5-μL aliquot of microdialysate was added to a screw-top glass GCMS vial containing 5 ng of dl-2HG-d_3_ internal standard in MeOH. The samples were vortexed and dried under a stream of nitrogen at 30°C. The samples were derivatized with DATAN according to a previously published procedure (18). The derivatized samples were resuspended in 50 μL of milli-Q water and analyzed by LC-MS/MS.

#### Plasma, Tumor Homogenate, and Brain Homogenates

Plasma samples were diluted 1:1 in milli-Q water. Tumor and brain homogenate samples were diluted to a final volume of 4 and 100 mg/mL, respectively, with milli-Q water. An 8-μL aliquot of diluted plasma, brain homogenate, or tumor homogenate was added to 32 μL of ice cold MeOH containing 75 ng/mL dl-2HG-d_3_ internal standard. The sample was vortexed and centrifuged at 14,000 × *g* for 10 min at 4°C. A 25-μL aliquot of the supernatant was added to a screw-top glass GCMS vial, evaporated under a stream of nitrogen gas, and derivatized as above. The derivatized sample was resuspended in 50 μL of milli-Q water and analyzed by LC-MS/MS.

#### Instrumentation and LC Parameters

Liquid chromatography–mass spectrometry was performed on a QTRAP 5500 triple quadrupole mass spectrometrer (SCIEX, Framingham, MA, USA) equipped with a Shimadzu Prominence UFLC liquid chromatography system (Shimadzu, Kyoto, Japan). A 5 μL of sample was injected onto a Luna C18(2) 50 mm × 2 mm, 3 μm column (Phenomenex, Torrance, CA, USA) and chromatographic separation occurred in water with 5 mM ammonium formate adjusted to pH 3.15 ± 0.05 with formic acid and 5% MeOH mixed online with a flow rate of 200 μL/min. The first 4 min of post-column eluent containing excess DATAN was diverted to waste to avoid contamination of the mass spectrometer source. After 4 min of chromatographic separation, post column eluent was introduced by electrospray ionization (ESI) into the mass spectrometer and monitored by multiple reaction monitoring (MRM) in negative ionization mode. Compound and instrument-dependent parameters were optimized for each compound of interest (see Supplementary Material).

#### Quantification of 2HG

For quantification of D2HG and L2HG in microdialysis samples, a standard curve from 20 to 10,000 ng/mL of D2HG and L2HG was created in CNS perfusion fluid and derivatized as above. Plasma, brain homogenates, and tumor homogenates were quantified using the isotope dilution method with standard curves in milli-Q water containing 20–300, 100–10,000, or 20–10,000 ng/mL D2HG and L2HG for plasma, tumor homogenates, and brain homogenates, respectively. The MRM transitions monitored were 363 → 147 for D2HG and L2HG and 366 → 150 for dl-2HG-d3 in microdialysis samples. Due to matrix interference, an MRM of 363 → 129 was used for L2HG in plasma and an MRM of 366 → 131 was used for dl-2HG-d3 in plasma, brain, and tumor homogenate samples. Data were processed and standards were fit to a 1/×-weighted linear regression using Analyst 1.6.2. Intraday and interday (*n* = 3) validation was performed for all matrices will replicates (*n* = 5) of low, medium, and high quality control standards (see Supplementary Material).

### Brain Distribution Model of D2HG

The compartmental model used for D2HG simulations specifies three subcompartments – intracellular interstitial fluid and CSF – for both the glioma and normal cell compartments (see Results). This is consistent with the physiologically based pharmacokinetic models of tissue distribution of intracellular, interstitial fluid, and vascular subcompartments; however, a brain vascular compartment was not considered as an important aspect of the current model for D2HG since plasma concentrations are minimal, about 1 μM, and would not recycle across the blood–brain barrier in significant amounts. The model only considers the two most relevant cell spaces; the glioma cells and those normal cells in close proximity that would most likely be exposed to D2HG in the adjacent interstitial fluid compartment. The model also includes a terminal CSF compartment that represents the subarachnoid spaces that do not exchange material with the brain interstitial fluid compartments (19, 20). The model differential equations and parameters are provided in Table S5 in Supplementary Material. Physiological volumes were calculated from standard relationships based on an average measured tumor weight of 100 mg and assuming a density of 1 g/mL. The CSF bulk flow rate was taken from the literature (21), and the brain interstitial fluid flow (6.0 × 10^−7^ L/h) was an intermediate value in the range of 0.15–0.29 μL/min/g estimated in rats (22). Other than the influx rate of D2HG into the intracellular compartments, which was considered to be non-linear, all intercompartmental clearances and the systemic clearance from plasma are considered to be linear or concentration independent. The non-linear intracellular influx was represented as a Michaelis–Menten transport process based on the likely mechanism that NaDC3 is a key dicarboxylic acid transporter (23, 24). The model rate equations and parameters are provided in the Supplemental Material.

#### Culture of Human T Cells, Intracellular Staining, and Flow Cytometric Analysis

Peripheral blood (PB) from de-identified healthy donors was collected in this study after obtaining informed consent. This study was approved by the IRB committee of Temple University. Naïve CD4^+^ T cells and CD8^+^ T cells were obtained from PB mononuclear cells (PBMCs) through negative selection using human naïve T cell isolation kit II from Miltenyi Biotec (San Diego, CA, USA). T cells were activated by anti-CD3 Ab + anti-CD28 Ab at day 0. D2HG – ranging from 0 to 500 μM concentrations – was added to the culture on day 0. Cells were collected at different time points of culture, counted, and analyzed for intracellular staining of IFN-γ using an intracellular staining kit from BD (BD Cytofix/Cytoperm™ Kit, BD Biosciences, CA, USA) and antibodies from Biolegend. Multi-color flow cytometric analysis was performed using a BD LSRII flow cytometer as described (25).

## Results

### D2HG Is Present in Micromolar Concentrations in Tumor Interstitial Fluid

**Figure 1:**
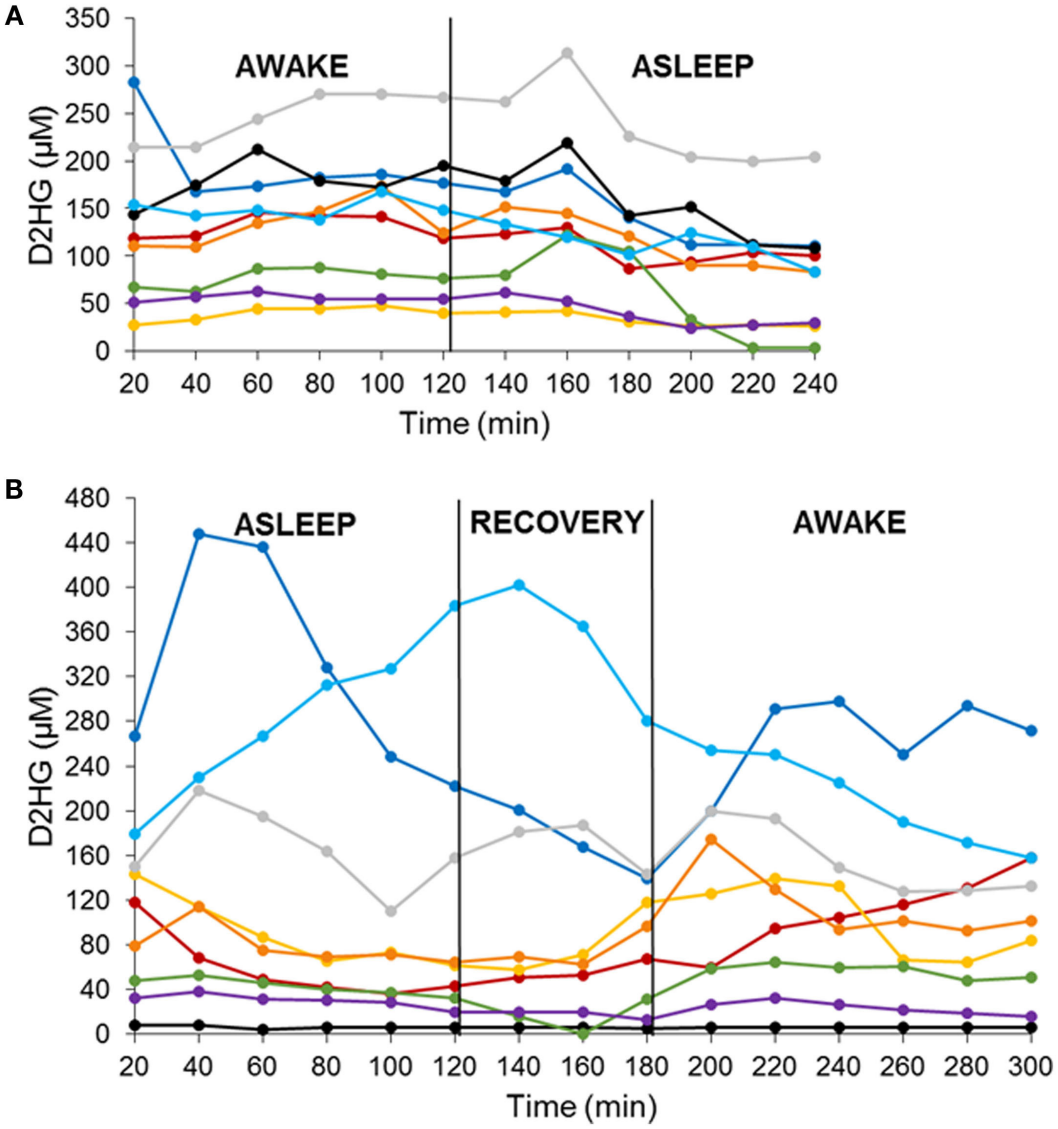
**D2HG brain tumor interstitial fluid concentrations in mIDH1/U87 tumor-bearing mice in two sequenced cohorts**. Awake–asleep **(A)** and asleep–awake **(B)**. Each line is representative of one mouse.

There were two cohorts of nine mice each studied in a crossover fashion with either an awake–asleep cycle or an asleep–awake cycle. Each awake and asleep study period was of a 2-h duration – 6 microdialysis samples – that included an equilibration period of 1-h prior to the start of the study and a 1-h re-equilibration period in the asleep–awake cohort to ensure the mice were fully conscious prior to the 2-h awake period sample collection. The concentrations of both D2HG and L2HG were monitored in BTIF by microdialysis in mice bearing intracerebral mIDH1/U87 brain tumors. Concentrations of L2HG were found to be under the lower limit of quantitation (1.35 μM) in all microdialysis samples collected. D2HG concentrations were measured in all microdialysis samples from each of the 18 mice (Figures 1A,B), wherein it can be seen that BTIF concentrations varied considerably between the mice with a mean ± SD of 122.3 ± 84.2 μM for all mice included in the study.

**Figure 2:**
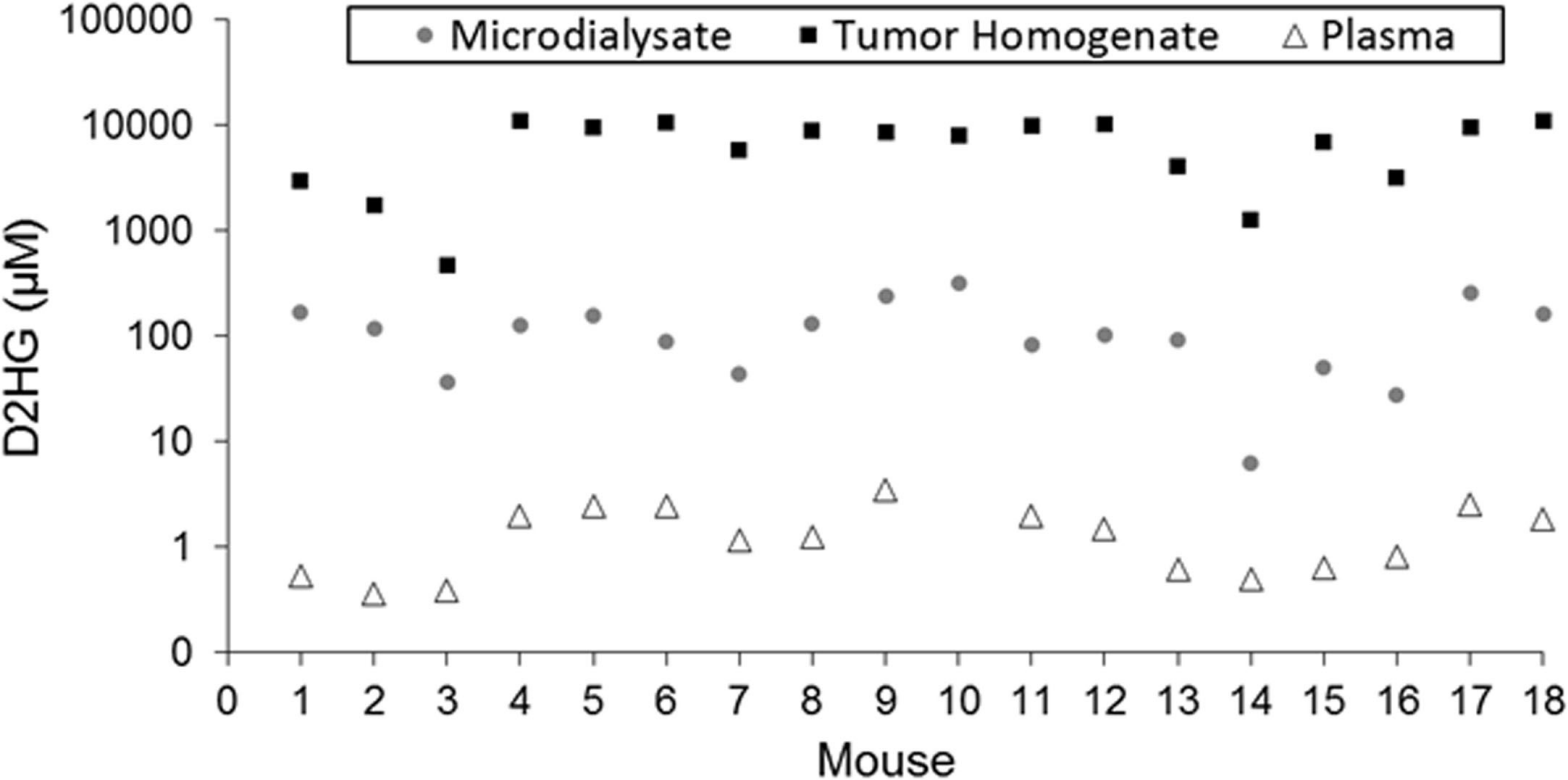
**Comparison of D2HG concentrations in microdialysate, tumor homogenate, and plasma**. Tumor homogenate concentrations were calculated assuming a tissue density of 1.00 mg/mL. Microdialysate values represent the average of the awake and asleep states after statistical analysis to determine steady-state segments in each period.

In order to analyze the D2HG BTIF concentrations, we defined a steady-state period to be the longest consecutive time period (a minimum of three samples or a 1-h period) of measured concentrations with a CV ≤ 25%. All 18 mice contained segments in both the awake and asleep states that fulfilled the steady-state inclusion criteria (Figure 2; Table 1). There was no sequence effect observed between the two cohorts (*p* = 0.98), indicative that the timing of anesthesia did not have an effect on the D2HG concentrations in the two states. Thus, there were a total of 36 study periods – 18 awake and 18 asleep – that produced mean D2HG BTIF concentrations in the asleep state of 117.6 versus 126.9 μM in the awake state.

**Table 1:** **Concentrations of D2HG in BTIF (steady-state) and brain tumor**.

		D2HG in BTIF (μM)				D2HG in tumor (μM)	Average BTIF/tumor (%)
Cohort	Mouse #	Awake	Asleep	Average	% Change		
Awake–asleep	1	194.6	139.1	166.8	−28.5	2912	5.7
	2	131.4	106.5	118.9	−18.9	1755	6.8
	3	39.4	32.5	36.0	−17.5	473	7.6
	4	133.4	119.6	126.5	−10.3	10,752	1.2
	5	179.3	128.8	154.1	−28.2	9427	1.6
	6	76.9	102.1	89.5	32.7	10,558	0.8
	7	56.0	29.5	42.7	−47.4	5832	0.7
	8	149.6	112.1	130.9	−25.1	8659	1.5
	9	246.7	234.7	240.7	−4.9	8473	2.8
Asleep−–awake	10	267.4	369.7	318.6	38.2	8043	4.0
	11	120.9	42.6	81.8	−64.8	9756	0.8
	12	132.7	71.8	102.3	−45.9	10,254	1.0
	13	104.1	79	91.5	−24.1	4076	2.2
	14	6.2	6.4	6.3	3.0	1256	0.5
	15	57.0	42.7	49.9	−25.1	6870	0.7
	16	25.2	30.3	27.7	20.2	3190	0.9
	17	208.4	304	256.2	45.9	9402	2.7
	18	155.2	165.9	160.5	6.9	10,845	1.5

### Concentration of D2HG Found in Brain Tumor Homogenate

Table 1 shows the concentrations of D2HG in total brain tumor homogenates, wherein it can be seen the values were in the millimolar range (assuming a tumor density of 1.00 g/mL), which is consistent with previous studies analyzing D2HG levels in R132H_mIDH1/U87 tumors (4, 26). The mean D2HG brain tumor concentration for all mice was 6.8 ± 3.6 mM, with a range from ~500 μM to >10 mM. No tumors contained quantifiable L2HG (LLOQ = 170 μM). The concentrations of D2HG in BTIF ranged from 0.5 to 7.6% (average = 2.4%) of the total tumor concentrations, and thus, quite variable between mice (Table 1). The tumor D2HG concentrations did not vary significantly between cohorts (*p* = 0.76), indicative that anesthesia did not impact the overall D2HG concentration in the tumors. The inter-animal variability in D2HG BTIF and tumor concentrations is likely due to the inherent heterogeneity of the tumors caused by regions of necrosis and proliferation that would affect the number of viable cells producing D2HG. In addition, the tumor homogenate concentrations reflect distribution into normal cells and its subpopulations that may be subjected to differential distribution. Finally, the heterogeneity is dependent on the actual collected sample; mIDH1/U87 tumors are well demarcated, so gross dissection to yield a tumor sample is not likely to introduce large errors; however, microdialysis sampling is dependent on the tumor environment and the restrictions imposed by the spatial resolution of microdialysis probe (27). Previous studies utilizing U87MG orthotopic xenograft models have displayed a heterogenous population of tumor cell types (28) as well as variable metabolic profiles among various tumor cells, usually correlated with hypoxic versus normoxic regions of tumors (29, 30).

### Concentrations in Ipsilateral/Contralateral Brain and Plasma

The concentrations of D2HG were quantifiable (≥1.35 μM) in all non-brain tumor samples. The concentrations (assuming a density of brain tissue of 1.00 g/mL) of D2HG in ipsilateral and contralateral brain ranged from 4.2 μM to 2 mM and 3.0 to 48.6 μM, respectively (Table S4 in Supplementary Material). In contrast, the L2HG levels in ipsilateral and contralateral brain ranged from 1.35 to 10.63 μM and 1.35 to 14.65 μM, respectively; however, concentrations in four ipsilateral and three contralateral brain samples were less than the lower limit of quantitation (1.35 μM).

Plasma samples collected from each mouse (excluding mouse 10, for which a usable sample volume could not be obtained) contained both isomers of 2HG in quantifiable concentrations (≥0.27 μM). The plasma concentrations of D2HG (Figure 2; Table S4 in Supplementary Material) and L2HG ranged from 0.36 to 3.48 μM and 0.41 to 1.44 μM, respectively. Although plasma D2HG concentrations were very low compared with tumor concentrations, there was a strong positive correlation observed between them (*r* = 0.78, *p* < 0.001), suggesting that tumor D2HG distributes from the CNS into the systemic circulation.

### Implications of D2HG Distribution in Brain Tumors

**Figure 3:**
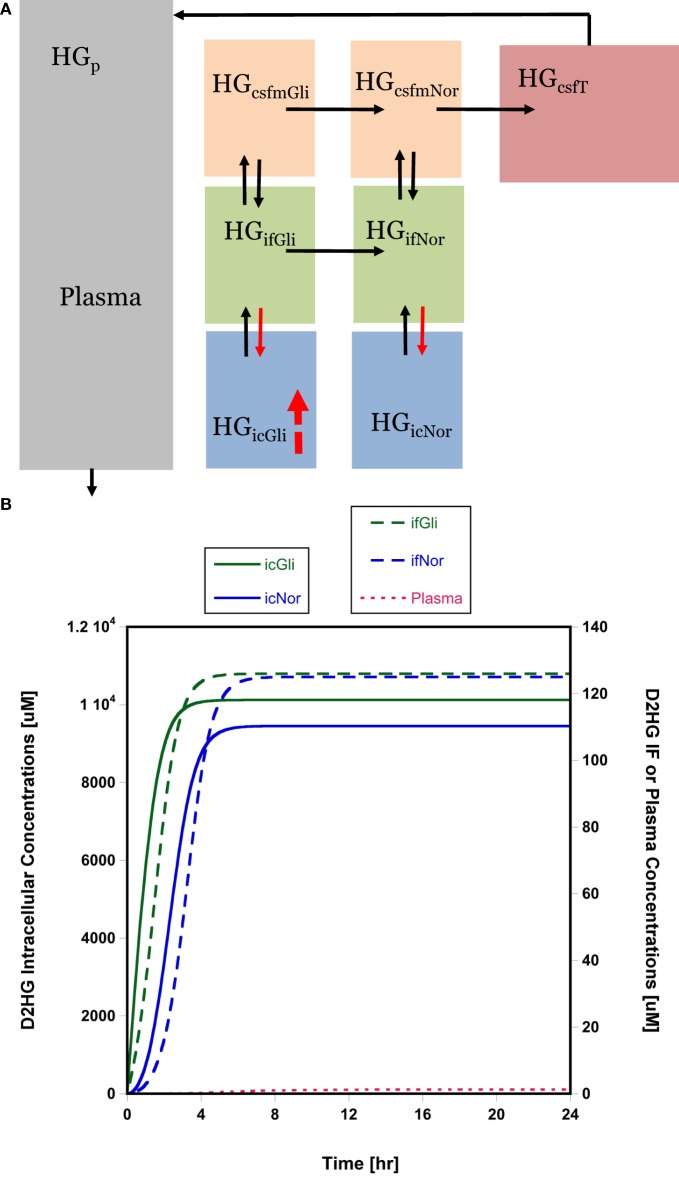
**Brain distribution model for D2HG**. **(A)** Model of D2HG (HG in figure) brain distribution. Each compartment is assumed to be homogeneous with respect to D2HG concentrations. Compartments (subscripts): icGli, intracellular glioma cells; icNor, intracellular normal cells; ifGli, interstitial fluid glioma; ifNor, interstitial fluid normal; csfmGli, main CSF glioma; csfmNor, main CSF normal; csfT, terminal CSF; p, plasma. Black arrows represent linear intercompartmental clearances, solid red arrows represent Michaelis–Menten influx *via* NaDC3 transporter, hatched red arrow represents zero-order production rate of D2HG. **(B)** Simulated D2HG concentrations in glioma and normal cell compartments using brain distribution model. Please note different *y*-axes scales. Abbreviations in the legends are the same as in **(A)**. Model equations and parameter values (Table S5 in Supplementary Material) are provided in Supplementary Material.

The high D2HG concentrations in total tumor homogenate bated the question of the cellular localization of D2HG. It was surmised that the high concentrations were not likely confined to glioma cells and might accumulate in normal cells, particularly those in the adjacent microenvironment, using interstitial fluid as a conduit for cell-to-cell transfer. This general phenomenon has been considered previously (22), and within the guise of D2HG being tumorigenic, it is fair to assume its uptake into normal glial or precursor cells and cells needed for an immune response could be a contributing factor. A semi-mechanistic model of D2HG was developed (Figure 3A, see Materials and Methods) that consisted of an eight-compartment model with intracellular, interstitial fluid, and CSF compartments for both the glioma and normal cell compartments. The model parameters included physiological volumes and brain interstitial and CSF fluid flow rates with the remaining transport parameters calibrated to the observed D2HG concentrations in the brain tumor, BTIF, and plasma; being about 6000, 125, and 1.5 μM, respectively. The total tumor homogenate D2HG concentration represents an intracellular glioma cell compartment concentration of 10,000 μM (see Materials and Methods). The model assumed linear intercompartmental transfer of D2HG except for the influx from interstitial fluid – both in the glioma and normal cell compartments – to the intracellular compartment where Michaelis–Menten transport was assumed based on the likelihood D2HG was a substrate for NaDC3 (SLC13A3) (23, 24). The simulations show (Figure 3B) that appreciable D2HG concentrations – 9400 μM – may be attained at steady state in the adjacent normal cell intracellular compartment or 93% of those attained in the glioma intracellular compartment. It can be seen that there is a time lag for each compartment to achieve steady-state D2HG concentrations consistent with a concentration gradient from the glioma interstitial fluid compartment to the normal cell interstitial fluid compartment due to the convective flux of D2HG once it exits glioma cells into the interstitial fluid space. As a simulation exercise there is uncertainty in the parameter values and other parameter sets provide analogous simulated results; nonetheless, the results support the feasibility that high D2HG concentrations in glioma cells permit flux into the interstitial fluid compartment for transfer into normal cells. This possibility prompted an investigation of D2HG action on immune cells, both CD4 and CD8 T-cells.

### D2HG Action on Immune Cells

**Figure 4:**
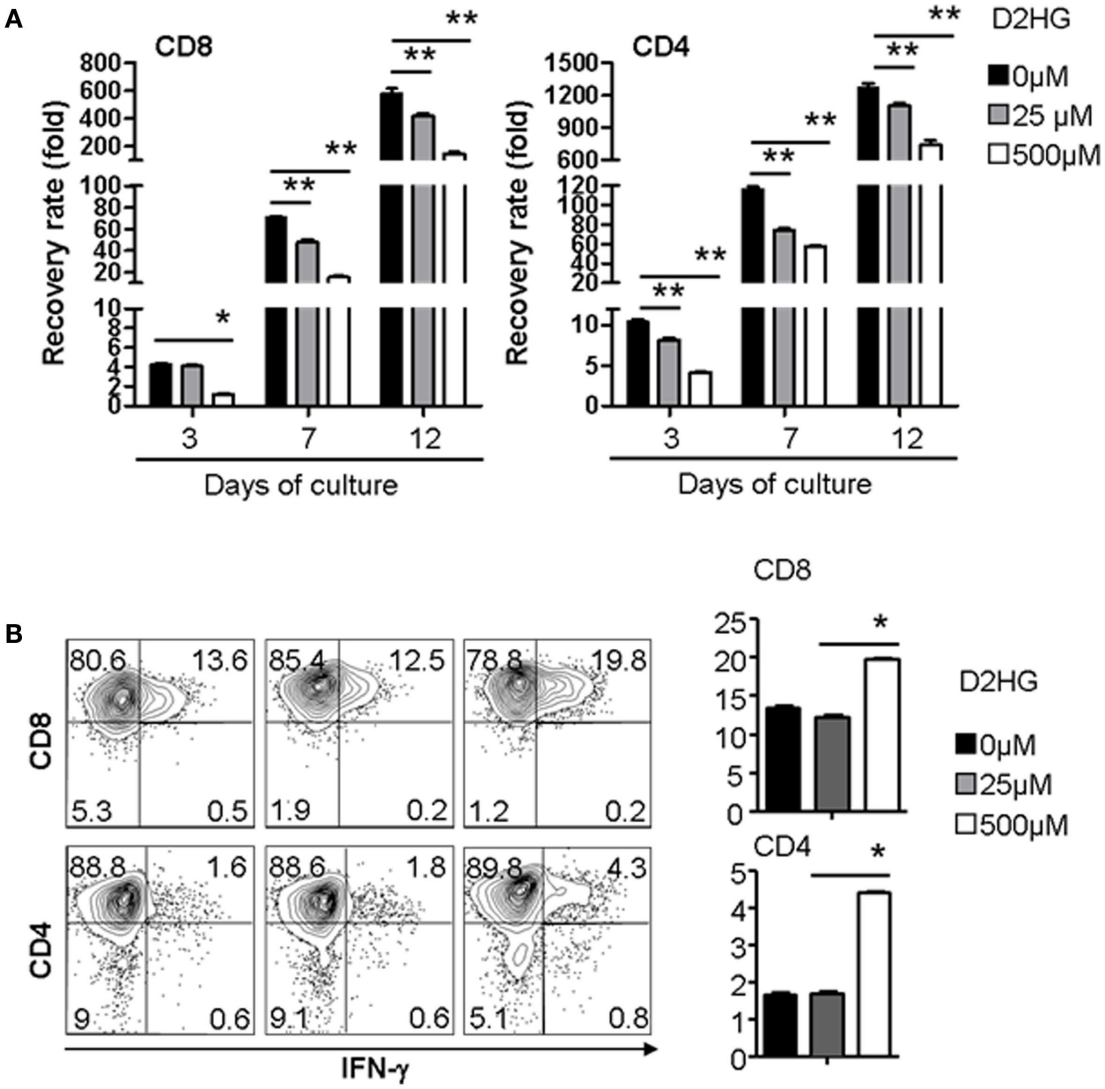
**D2HG reduces proliferation of human T cells cultured *in vitro***. Human CD4 and CD8 T cells were activated by anti-CD3 Ab + anti-CD28 Ab at day 0. A range of D2HG concentrations was added to the culture on day 0. **(A)** At the indicated time points, the number of T cells in the culture was counted, calculated for the recovery rate based on the following formula: fold change of recovery rate = output cell number/input cell number. **(B)** Cells were collected at day 12 for intracellular staining of IFN-γ and flow cytometry analysis (*p < 0.05).

To test the possible action of D2HG on immune cells, we examined the effect of D2HG on the proliferation and IFN-γ by human T cells that were activated by anti-CD3 antibody (Ab) and anti-CD28 Ab. Addition of D2HG caused a significant decrease (twofold to threefold) in expansion of these activated CD4^+^ and CD8^+^ T cells in a dose-dependent manner (Figure 4A). Interestingly, treatment with 500 μM D2HG enhanced the production of IFN-γ-producing T cells in cultures compared with untreated controls (Figure 4B). These results suggest that glioma cell-derived D2HG may interfere with T cell immune function.

## Discussion

Patients with mutant IDH1 brain tumors are prevalent with a number of investigations describing the biological and biochemical effects of the oncometabolite, D2HG, which is produced in high quantities. Among the panoply of D2HG actions are its ability to inhibit KG-dependent enzymes and to remodel the methylome creating the G-CIMP phenotype (7). The consequences of these effects are unanswered as are questions related to the brain disposition of D2HG and whether its compartmentalization in the brain and brain tumor might suggest how it may be involved with tumorigenesis and whether alternate and companion treatments to mutant IDH1 inhibitors could play a role.

This investigation provides the first direct evidence that D2HG exits glioma cells and is present in the interstitial fluid compartment at micromolar concentrations. The D2HG concentrations are a small fraction of those measured in total tumor homogenates, and on face value suggests that therapeutic modalities directed at BTIF D2HG may be unwarranted. One BTIF-directed approach – glymphatic clearance activation – produced an average 7% reduction in D2HG steady-state concentrations under anesthesia, and even though one could envision that pharmacological means to accelerate glymphatic clearance over a longer duration could be more effective than the 2-h asleep periods evaluated here it is not likely to materialize into a major effect. Nonetheless, there are other scenarios where BTIF-directed therapy may be effective and could be combined with mIDH1 inhibitors. The BTIF D2HG concentrations provide a pathway for D2HG to enter normal cells and contribute to tumorigenesis. Of the normal cell types that D2HG could adversely affect are cells involved in the immune response, both T-cells and dendritic cells. For D2HG to be effectively taken up into normal cells, such as T-cells, there are likely one or more membrane transport systems. As a dicarboxylic acid it is likely D2HG is a substrate for SLC family members that transport KG, and in fact, D2HG was a substrate of NaDC3 (SLC133A) (*K*_m_ = 164 ± 14 μM) in oocytes that overexpressed it (31). Additional studies in cell systems and including other carrier proteins seem warranted. These membrane transport proteins function in either mono- or bi-directional capacities and could be an alternate target to prevent cell uptake. Thus, one hypothesis is D2HG is effluxed from glioma cells into BTIF space, wherein it is available to enter normal cells *via* NaDC3 or other carriers and accumulate to high concentrations. To test the feasibility of this hypothesis, we developed a model for D2HG (Figure 3A) brain disposition that consisted of intracellular, interstitial fluid, and CSF glioma and normal cell compartments connected *via* bulk flow of interstitial fluid. The simulated results – although not confirmed – indicate appreciable D2HG distribution into the normal cell intracellular compartment. This observation raises the specter of how D2HG exerts its tumorigenic action and supports studies to dissect its action in specific cell types rather than whole tumor homogenate analyses.

Given the high relevance of immune function in cancer, we considered immune cells as possible normal cell recipients of D2HG. Recent studies have demonstrated that the tumor microenvironment critically influences antitumor activity of T cells. Tumor cells may deplete nutrients essential for T cell survival and expansion, leading to impaired T cell immunity against tumor (32). Our findings suggest another important mechanism by which tumors may reduce anti-tumor immunity. Glioma cells produce high levels of D2HG that permeate the local environment and cause significant reduction of T cell expansion and persistence, impairing antitumor effect of these T cells. In addition, some studies have demonstrated that although IFN-γ is important for T cell-mediated antitumor immunity (33), it also causes increased tumor cell expression of PD-L1, which is an immune-check point inhibitor. It is possible that increased production of IFN-γ by glioma cell-derived D2HG might contribute to the upregulation of PD-L1 in tumors, thereby further impairing T cell antitumor activity. Thus, our findings indicate that inhibition of D2HG – although generally sought – may represent a new strategy to improve tumor immunotherapy.

## Author Contributions

AP developed LC/MS/MS methods and obtained associated data, designed experiments, assisted AS and TB with assays, and helped to write paper. AS developed mouse techniques with JG and performed all mouse experiments, including tumor cell methods, helped process samples, and wrote pertinent methods sections for manuscript. TB either assisted both AS and AP on all assays or performed independently including mouse sample collections and running LC/MS/MS. SH and YZ developed and completed all *in vitro* cell assays related to immune function, and wrote appropriate sections for papers. JG developed over-arching hypotheses related to studies, designed experiments with AP and AS, analyzed data and developed CNS model for D2HG and completed model simulations and wrote paper with AP along with contributions from others.

## Conflict of Interest Statement

The authors declare that the research was conducted in the absence of any commercial or financial relationships that could be construed as a potential conflict of interest. The reviewer AZ and handling editor declared their shared affiliation, and the handling editor states that the process nevertheless met the standards of a fair and objective review.
