# Supplementary material for: Intracerebral Distribution of the Oncometabolite d-2-Hydroxyglutarate in Mice Bearing Mutant Isocitrate Dehydrogenase Brain Tumors: Implications for Tumorigenesis
Source: Front Oncol. 2016 Oct 11;6:211. doi: 10.3389/fonc.2016.00211 (PMC5057413; doi:10.3389/fonc.2016.00211)
Supplement: Supplementary file 1 [file Data_Sheet_1.DOCX]

**Supplementary Information: Intracerebral Distribution of the Oncometabolite D-2-Hydroxyglutarate in Mice Bearing Mutant Isocitrate Dehydrogenase Brain Tumors: Implications for Tumorigenesis**

Amanda J. Pickard^1^, Albert S. W. Sohn^1^, Thomas F. Bartenstein^1^, Shan He^2^, Yi Zhang^2^, James M. Gallo^1^

^1^Department of Pharmacology and Systems Therapeutics, Icahn School of Medicine at Mount Sinai, New York, NY. ^2^Fels Institute for Cancer Research and Molecular Biology, ^‡^Department of Microbiology and Immunology, Temple University, Philadelphia, PA

Table of Contents

1. Optimization of Compound-dependent Mass Spectrometer Parameters
2. **Table S1**: Compound-dependent mass spectrometer parameters
3. Optimization of Ion Source-dependent Mass Spectrometer Parameters
4. Calibration of Microdialysis Probes
5. Method Validation in Microdialysate, Plasma, Brain Homogenate and Tumor Homogenate
6. **Table S2**: Precision and accuracy data for the analysis of 2HG
7. Calibration of Microdialysis Probes
8. **Table S3:** Recovery of D2HG during Probe Calibration Studies
9. **Table S4:** D2HG Concentrations in Plasma and Brain Homogenates
10. CNS Distribution Model for D2HG
11. **Table S5:** Parameters for D2HG brain distribution model

*Compound-dependent Mass Spectrometer Parameters:*

A derivatized sample of D2HG or DL-2HG-d3 was diluted to 1 µM in 50% MeOH and infused into the mass spectrometer at a flow rate of 7 µL/min with an integrated syringe pump. Declustering potential (DP), entrance potential (EP), collision energy (CE) and collision cell exit potential (CXP) were optimized for each compound (Table S1). Each MRM transition had a dwell time of 150 ms.

**Table S1: Compound-dependent Mass Spectrometer Parameters**

| **Transition** | **Q1 (m/z)** | **Q3 (m/z)** | **DP** | **EP** | **CE** | **CXP** |
| --- | --- | --- | --- | --- | --- | --- |
| 2HG_1 | 362.832 | 147.100 | -60 | -2 | -20 | -12 |
| 2HG_2 | 362.832 | 128.800 |  |  |  |  |
| 2HG-d3_1 | 365.832 | 150.000 |  | -4 | -40 | -36 |
| 2HG-d3_2 | 365.832 | 131.800 |  |  |  |  |

*Ion Source-dependent Mass Spectrometer Parameters:*

A 10 µM solution of derivatized D2HG was prepared in milli-Q water. 5 µL of this solution was introduced into the mass spectrometer in 95% 5 mM NH3COOH (pH 3.15) and 5% MeOH at a flow rate of 0.200 mL/min by direct injection. Curtain gas (CUR), collision-activated dissociation gas (CAD), ion transfer voltage (IS), auxiliary gas temperature (TEM), nebulizer gas (GS1) and auxiliary gas (GS2) were optimized by flow injection analysis. The optimized parameters were as follows: CUR, 40 psi; CAD, Medium; IS, -4500 V; TEM, 500°C; GS1 80 psi, GS2 50 psi.

*Method Validation in Microdialysate, Plasma, Brain Homogenate and Tumor Homogenate*

Intraday and interday (n=3) validation was performed for all matrices will replicates (n=5) of low, medium, and high quality control standards. Endogenous amounts of D2HG and L2HG were determined in each matrix and subtracted from the total amounts found to determine the concentration of the spiked sample. Intraday and interday coefficients of variation (CV) were determined by one-way ANOVA. Validation criteria included intra/interday CVs of ≤15%, recovery +/- 15% nominal value, standard curve linearity ≥ 99.5%, and standard curve samples +/- 15% nominal value (+/- 20% for LLOQ). Validation results are presented in **Table S2**.

**Table S2: Precision and Accuracy Data for the Analysis of 2HG**

| **Matrix** | **Standard Curve (ng/mL)** | **Analyte (n=5)** | **Concentration (ng/mL)** | | **CV** | | **Recovery** |
| --- | --- | --- | --- | --- | --- | --- | --- |
|  |  |  | **Nominal** | **Found** | **Intra-day** | **Inter-day**  **(n = 3)** |  |
| aCSF | 20-10,000 | D2HG | 75 | 77 | 7.9% | 1.3% | 102.2% |
|  |  |  | 750 | 759 | 9.5% | 14.5% | 101.2% |
|  |  |  | 7500 | 7603 | 6.2% | 6.3% | 101.4% |
|  |  | L2HG | 75 | 76 | 6.4% | 2.4% | 101.9% |
|  |  |  | 750 | 762 | 7.2% | 14.3% | 101.6% |
|  |  |  | 7500 | 7484 | 6.9% | 11.2% | 99.8% |
| Plasma | 20-300 | D2HG | 80 | 78 | 6.6% | 11.1% | 96.92% |
|  |  |  | 160 | 146 | 4.7% | 1.9% | 91.25% |
|  |  |  | 240 | 230 | 5.6% | 8.7% | 95.89% |
|  |  | L2HG | 80 | 77 | 8.6% | 9.1% | 95.86% |
|  |  |  | 160 | 151 | 6.0% | 9.3% | 94.10% |
|  |  |  | 240 | 228 | 7.2% | 3.1% | 94.93% |
| Brain | 20-10,000 | D2HG | 75 | 73 | 8.6% | 11.7% | 97.99% |
|  |  |  | 750 | 727 | 2.4% | 2.8% | 96.96% |
|  |  |  | 7500 | 7668 | 3.8% | 7.3% | 102.25% |
|  |  | L2HG | 75 | 69 | 6.4% | 14.8% | 92.53% |
|  |  |  | 750 | 692 | 3.3% | 3.1% | 92.25% |
|  |  |  | 7500 | 7390 | 3.6% | 6.8% | 98.53% |
| Tumor | 100-10,000 | D2HG | 750 | 795 | 4.9% | 11.3% | 105.9% |
|  |  |  | 7500 | 7938 | 5.6% | 4.6% | 105.8% |
|  |  | L2HG | 750 | 780 | 4.9% | 7.1% | 104.0% |
|  |  |  | 7500 | 7844 | 4.8% | 5.3% | 104.6% |

*Calibration of Microdialysis Probes*

The microdialysis probes were flushed with 70% ethanol, water, and artificial CSF prior to equilibration. The probe was then placed in a 200 µL polypropylene tube (reservoir) containing 150 µL of CSF with 100, 200, or 300 µM D2HG. The probe was perfused for 20 minutes at 0.5 μL/min for sterile water and 0.5 μL/min for artificial CSF. Flow rates were changed to 0.9 μL/min for sterile water and 0.1 μL/min for artificial CSF for an additional 60 minutes of equilibration prior to collecting the calibration samples. The calibration samples were collected in 20 minute intervals for 80 minutes into a CMA (Harvard Apparatus, Holliston, MA) 470 fraction collector that maintained the samples at 4-6°C for the duration of the study.

For quantification of D2HG in calibration samples, a standard curve from 50-100,000 ng/mL of D2HG was created in water. A 5 µL aliquot of standards, quality control samples, and microdialysis samples were added to 45 µL of ice-cold MeOH containing 200 ng/mL ^13^C_5_-D2HG. The samples were vortexed and centrifuged at 14,000xG for 10 minutes at 4°C. A 40 µL aliquot of the supernatant was added to a 96 deep well plate.

Liquid-chromatography mass spectrometry was performed on a QTRAP 5500 triple quadrupole mass spectrometry (SCIEX, Framingham, MA) equipped with a Shimadzu Prominence UFLC liquid chromatography system (Shimadzu, Kyoto, Japan). 1 µL of sample was injected onto a Synergi Hydro-RP 100×2 mm, 2.5 µm column (Phenomenex, Torrance, CA) and chromatographic separation occurred in water 0.1% formic acid at a flow rate of 200 µL/min. Post column eluent was introduced by electrospray ionization (ESI) into the mass spectrometer and monitored by multiple reaction monitoring (MRM) in negative ionization mode. The mass spectrometer parameters were as follows: CUR, 45 psi; CAD, Low; IS, -4500 V; TEM, 650°C; GS1 75 psi, GS2 35 psi. The MRM transitions monitored were 147 🡪 57 for D2HG and 152 🡪 60 for ^13^C_5_-D2HG. Data was processed and standards were fit to a 1/x-weighted linear regression using Analyst 1.6.2.

**Table S3: Recovery of D2HG during Probe Calibration Studies**

|  | **D2HG Concentration in Reservoir** | | | | | |
| --- | --- | --- | --- | --- | --- | --- |
|  | **100 µM** | | **200 µM** | | **300 µM** | |
| **Trial** | **Average** | **Std Dev** | **Average** | **Std Dev** | **Average** | **Std Dev** |
| **1** | 54.9% | 1.3% | 47.2% | 1.2% | 50.15% | 2.16% |
| **2** | 31.4% | 0.9% | 52.9% | 3.4% | 56.20% | 1.09% |
| **3** | 45.3% | 4.2% | 54.7% | 2.3% | 47.86% | 1.00% |
| **4** | 54.7% | 0.7% | 53.3% | 0.7% | 56.27% | 1.35% |
| **5** | N/A | N/A | N/A | N/A | 45.81% | 2.71% |
| **Average** | 46.6% | | 52.0% | | 51% | |
| **Std Dev** | 4.6% | | 4.3% | | 3.6% | |
|  | | | | | | |
| **Total Recovery** = 50 ± 7% | | | | | | |

**Table S4: D2HG Concentrations in Plasma and Brain Homogenates**

| **Cohort** | **Mouse #** | **D2HG (µM)** | | |
| --- | --- | --- | --- | --- |
|  |  | **Plasma** | **Ipsilateral Brain** | **Contralateral Brain** |
| **Awake-Asleep** | **1** | 0.54 | 4.15 | 4.78 |
|  | **2** | 0.36 | 6.46 | 7.26 |
|  | **3** | 0.38 | 4.92 | 5.51 |
|  | **4** | 1.94 | 48.00 | 8.47 |
|  | **5** | 2.40 | 137.40 | 17.28 |
|  | **6** | 2.39 | 14.21 | 13.27 |
|  | **7** | 1.15 | 158.33 | 41.66 |
|  | **8** | 1.22 | 53.30 | 16.10 |
|  | **9** | 3.48 | 1942.81 | 14.28 |
| **Asleep-Awake** | **10** | N/A | 10.36 | 6.08 |
|  | **11** | 1.95 | 13.23 | 9.38 |
|  | **12** | 1.47 | 315.31 | 19.72 |
|  | **13** | 0.62 | 177.91 | 15.36 |
|  | **14** | 0.50 | 7.29 | 7.93 |
|  | **15** | 0.64 | 6.41 | 5.27 |
|  | **16** | 0.82 | 15.53 | 16.04 |
|  | **17** | 2.56 | 149.21 | 48.61 |
|  | **18** | 1.82 | 24.58 | 9.93 |

*CNS Distribution Model for D2HG*

The ordinary differential equations for the model illustrated in Figure 3A are as follows:

Glioma Compartments:

$$\begin{matrix} V_{icGli}\frac{dHG_{icGli}}{dt}=HGsource+\left[\frac{V_{mGli}HG_{ifGli}}{K_{mGli}+HG_{ifGli}}\right]-CL_{icifGli}HG_{icGli} \\ V_{ifGli}\frac{dHG_{ifGli}}{dt}=CL_{icifGli}HG_{icGli}-\left[\frac{V_{mGli}HG_{ifGli}}{K_{mGli}+HG_{ifGli}}\right]-Q_{ifGliNor}HG_{ifGli}+CL_{csfmifGli}HG_{csfmGli}-CL_{ifcsfmGli}HG_{ifGli} \\ V_{csfmGli}\frac{dHG_{csfmGli}}{dt}=CL_{ifcsfm}HG_{ifGli}-CL_{csfmif}HG_{csfmGli}-Q_{csfmGliNor}HG_{csfmGli} \end{matrix}$$

Normal Cell Compartment:

$$\begin{matrix} V_{icNor}\frac{dHG_{icNor}}{dt}=\left[\frac{V_{mNor}HG_{ifNor}}{K_{mNor}+HG_{ifNor}}\right]-CL_{icifNor}HG_{icNor} \\ V_{ifNor}\frac{dHG_{ifNor}}{dt}=CL_{icifNor}HG_{icNor}-\left[\frac{V_{mNor}HG_{ifNor}}{K_{mNor}+HG_{ifNor}}\right]+Q_{ifGliNor}HG_{ifGli}+CL_{csfmifNor}HG_{csfmNor}-CL_{ifcsfmNor}HG_{ifNor} \\ V_{csfmNor}\frac{dHG_{csfmNor}}{dt}=CL_{ifcsfmNor}HG_{ifNor}-CL_{csfmif}HG_{csfmNor}+Q_{csfmGliNor}HG_{csfmGli}-Q_{csfmNorT}HG_{csfmNor} \\ \end{matrix}$$

CSF Terminal and Plasma Compartments:

$$\begin{matrix} V_{csfT}\frac{dHG_{csfT}}{dt}=Q_{csfmNorT}HG_{csfmNor}-Q_{csfTp}HG_{csfT} \\ V_{p}\frac{dHG_{p}}{dt}=Q_{csfTp}HG_{csfT}-CL_{p}HG_{p} \end{matrix}$$

**Table S5: Parameters for D2HG brain distribution model**

| Parameter | Value [units] | Definition |
| --- | --- | --- |
| VicGli | 3.5e-5 [L] | volume of the intracellular (IC) glioma compartment |
| VifGli | 1.5e-5 [L] | volume of the interstitial fluid (IF) glioma compartment |
| VcsfGli | 4.9e-6 [L] | volume of the CSF glioma compartment |
| VicNor | 3.5e-5 [L] | volume of the IC normal cell compartment |
| VifNor | 1.5e-5 [L] | volume of the IF normal cell compartment |
| VcsfNor | 4.9e-6 [L] | volume of the CSF normal cell compartment |
| VcsfT | 5.4e-5 [L] | volume of the terminal CSF compartment |
| Vp | 7.5e-2 [L] | volume of the plasma compartment |
| CL_icifGli_ | 3.3e-3 [L/hr] | IC to IF D2HG efflux clearance in glioma compartment |
| CL_icifNor_ | 3.3e-3 [L/hr] | IC to IF D2HG efflux clearance in normal cell compartment |
| V_mGli_ | 45 [umoles/hr] | maximum transport rate from IF to IC glioma compartment |
| K_mGli_ | 45 [uM] | Michaelis constant from IF to IC glioma compartment |
| V_mNor_ | 45 [umoles/hr] | maximum transport rate from IF to IC normal cell compartment |
| K_mNor_ | 45 [uM] | Michaelis constant from IF to IC normal cell compartment |
| Q_ifGliNor_ | 6.0e-7 [L/hr] | IF bulk flow from glioma to normal cell compartment |
| HG_source_ | 0.25 [umole/hr] | zero-order synthesis rate of D2HG in glioma IC compartment |
| CL_ifcsfmGli_ | 0.25 [L/hr] | IF to CSF main clearance rate in glioma compartment |
| CL_csfmifGli_ | 2.5e-3 [L/hr] | CSF main to IF clearance rate in glioma compartment |
| CL_ifcsfmNor_ | 0.25 [L/hr] | IF to CSF main clearance rate in normal cell compartment |
| CL_csfmifNor_ | 2.5e-3 [L/hr] | CSF main to IF clearance rate in normal cell compartment |
| Q_csfmGliNor_ | 2.0e-5 [L/hr] | CSF main bulk flow rate from glioma to normal cell compartment |
| Q_csfmNorT_ | 2.0e-5 [L/hr] | CSF main bulk flow rate from normal cell to terminal CSF compartment |
| Q_csfTp_ | 2.0e-5 [L/hr] | terminal CSF bulk flow rate to plasma compartment |
| CLp | 2.0 [L/hr] | systemic clearance of D2HG |
